# Cross-cultural adaptation and validation of cognitive emotion regulation questionnaire: a systematic review

**DOI:** 10.3389/fpsyg.2024.1494665

**Published:** 2025-03-19

**Authors:** Fatemeh Fekar Gharamaleki, Zeinab Fathipour-Azar

**Affiliations:** ^1^Department of Speech Therapy, Tabriz University of Medical Sciences, Tabriz, Iran; ^2^Department of Occupational Therapy, Tabriz University of Medical Sciences, Tabriz, Iran

**Keywords:** assessment, cognition, emotional regulations, reliability, validity

## Abstract

**Background:**

The Cognitive Emotion Regulation Questionnaire (CERQ) is an important instrument for assessing the perceived effectiveness of emotional regulation strategies. It has been adapted into numerous regional languages worldwide. This systematic review seeks to explore the various versions of the CERQ, focusing on their processes of linguistic and cultural adaptation, as well as their validity and reliability.

**Methods:**

Articles were systematically extracted from the literature review using search engines such as PubMed, Google Scholar, Web of Science, and Scopus. The focus was on identifying studies published in English between the years 2000 and 2024. This review also encompassed various versions of the CERQ that had been adapted and validated to accommodate linguistic and cultural differences.

**Results:**

The original database yielded 1,476 search results. After filtering out duplicates, 420 articles were examined. Following a review of the titles and abstracts, 21 studies were identified for further evaluation. Ultimately, 13 versions were chosen for the final analysis.

**Conclusion:**

This review offers an in-depth insight into the difficulties faced in cross-cultural adaptation and the psychometric assessment processes. Notably, the predominant approach for translation identified in the reviewed literature was Brislin’s classic back-translation model. The findings demonstrate strong test–retest reliability, excellent internal consistency, and reasonable construct validity across various languages, affirming the usability of the translated versions of the CERQ in different linguistic contexts.

## Introduction

Cognitive emotion regulation (CER) involves a wide range of conscious and unconscious physiological, behavioral, and cognitive aspects and this concept has rapidly grown in the past two decades (Grecucci et al., 2020; Fathipour-Azar and Khalafbeigi, 2021). Emotion regulation is defined as strategies to maintain, increase, or suppress a current affective state and includes the ability to regulate emotions and physiological changes to respond to a situation adequately (Shahzad et al., 2022; Tyra et al., 2024; Kraft et al., 2024). Emotional regulation in individuals encompasses both adaptive and maladaptive strategies (Kraft et al., 2024; Prastuti et al., 2020; Fathipour-Azar and Hejazi, 2018). Cognitive emotion regulation can influence both social interactions and individual behavior due to its crucial role in managing thought processes (Prastuti et al., 2020). Research indicates that the ability to regulate emotions cognitively, combined with mindfulness, is a key factor in social cognition, particularly when it comes to comprehending others’ emotions and practicing perspective-taking (Abdi et al., 2012).

So far, several tools have been designed for emotional cognitive evaluation, one of the most important of which is the Cognitive Emotion Regulation Questionnaire (CERQ). The CERQ was originally developed by Garnefski et al. (2001) and colleagues using a sample of high school students in the Netherlands. The CERQ assesses individual cognitive strategies for regulating emotions in response to challenging life circumstances (Garnefski and Kraaij, 2007). By examining cognitive processes following negative or stressful experiences, the CERQ aims to illuminate how these processes influence emotional development over time (Garnefski et al., 2001). Comprising 36 items, the questionnaire includes four items corresponding to each of the nine dimensions of emotional regulation strategies: (Grecucci et al., 2020) self-blame; (Fathipour-Azar and Khalafbeigi, 2021) acceptance; (Shahzad et al., 2022) rumination; (Tyra et al., 2024) putting into perspective; (Kraft et al., 2024) positive refocus; (Prastuti et al., 2020) refocus on planning; (Fathipour-Azar and Hejazi, 2018) positive reappraisal; (Abdi et al., 2012) catastrophizing; and (Garnefski et al., 2001) blaming others (Santos et al., 2023). The questionnaire includes 9 subscales and each subscale consisting 4 items (Santos et al., 2023).

Most of these cognitive-emotional assessment tools were developed in English in the United States or the United Kingdom (Gorecki et al., 2014). To meet the needs of culturally and linguistically diverse populations, both nationally and internationally, these tools require translation. In recent years, numerous studies have worked to translate the CERQ to suit their specific languages and cultural frameworks (Kraft et al., 2024). As a result, the original English version of the CERQ has been translated and validated into multiple languages, including Brazilian Portuguese, Hungary, German, Arabic, Tunisian, Turkish, Spanish, and several other versions (Garnefski et al., 2001; Uzzaman et al., 2024; Miklósi et al., 2011; Tuna and Bozo, 2012; Domínguez-Sánchez et al., 2013; Saad and Kamel, 2020; Fekar Gharamaleki et al., 2023; Ouerchefani et al., 2021). This process of cross-cultural adaptation ensures that the CERQ accurately captures the diverse experiences of different populations, addressing potential biases that may arise during translation and interpretation. To achieve cultural equivalence, a systematic adaptation process, incorporating expert evaluations and pre-testing, is vital, ensuring that the modified tool remains both reliable and valid for its intended audience (Tomas et al., 2022).

The adaptation process is influenced by the linguistic concept of a specific language, as well as the cultural contexts of the community (Cycyk et al., 2021). Equivalence adaptation involves several steps, including sentence translation, modification, expert comment, and validation (Weir, 2005; Fekar Gharamaleki et al., 2024; Farmani et al., 2024; Bahrami and Fekar-Gharamaleki, 2021). To perform a standard adaptation, researchers need an in-depth knowledge of these processes and the methodological distinctions (Savin-Baden and Major, 2023). The results reveal that although the CERQ has been implemented in several countries, there is a substantial requirement for translation and validation in most languages. This review emphasizes that firstly, it is necessary to use the target language version for the questionnaire because the language is effective on a person’s understanding of the instrument items. Secondly, it is essential to utilize the correct method in translation. Therefore, invalid translations can affect the health assessment, so the researchers emphasize the importance of standardized approaches in future adaptations and validation. This study intends to examine the methodologies involved in the cross-cultural adaptation of CERQ across different languages and to offer recommendations for improvement. By synthesizing existing research on the cross-cultural adaptation and validation of CERQ in various languages, the review stresses the importance of methodological precision in translation and cultural relevance to bolster both validity and reliability. Ultimately, this article calls on researchers to prioritize thorough cross-cultural adaptation processes to enhance the global relevance of cognitive strategies related to emotion regulation assessments like CERQ.

## Materials and methods

### Literature search

For this systematic review, we included relevant literature from 2000 to 2024 that focused on translating and evaluating the psychometric properties of the CERQ.

### Search strategies

In this study, we conducted a systematic literature search using databases such as PubMed, Google Scholar, Web of Science, and Scopus. The search was based on the keywords (“Cognitive Emotion Regulation Questionnaire” OR “CERQ”) AND (“Cross-Cultural Adaptation” OR “Translation”) AND (“Validation” OR “Psychometric Properties” OR “Validity” OR “Reliability”). The present systematic review utilized the PRISMA checklist for conducting the study (Page et al., 2021).

### Methodological evaluation

Initially, we screened titles related to the mentioned specified keywords. Articles retrieved from each literature search database were exported as research information system (RIS) files and then imported into Covidence^1^ for abstract and full-text screening (Azeroual et al., 2019; Kellermeyer et al., 2018). Subsequently, two reviewers (authors) independently assessed abstracts and excluded the irrelevant articles. In cases of disagreement, a third reviewer was consulted to reach a consensus. He was not the author of the research. Following this, the reviewers thoroughly examined the full text of relevant articles and selected those aligned with the research title and aim. The psychometric properties of the instrument were meticulously examined by the COSMIN (consensus-based standards for the selection of health measurement instruments) guidelines. These guidelines provide a comprehensive framework for evaluating the quality of health measurement instruments, ensuring that the studies are both reliable and valid for their intended purposes.

### Eligibility criteria and data extraction

Psychometric studies conducted in English between 2000 and 2024 were included in the study. Observational studies randomized controlled trials (RCTs), cross-sectional studies, case reports, qualitative studies, protocol studies, review articles, and published abstracts were excluded. Full text of articles in non-English were excluded from the analysis.

## Results

### Study selection

**Figure 1:**
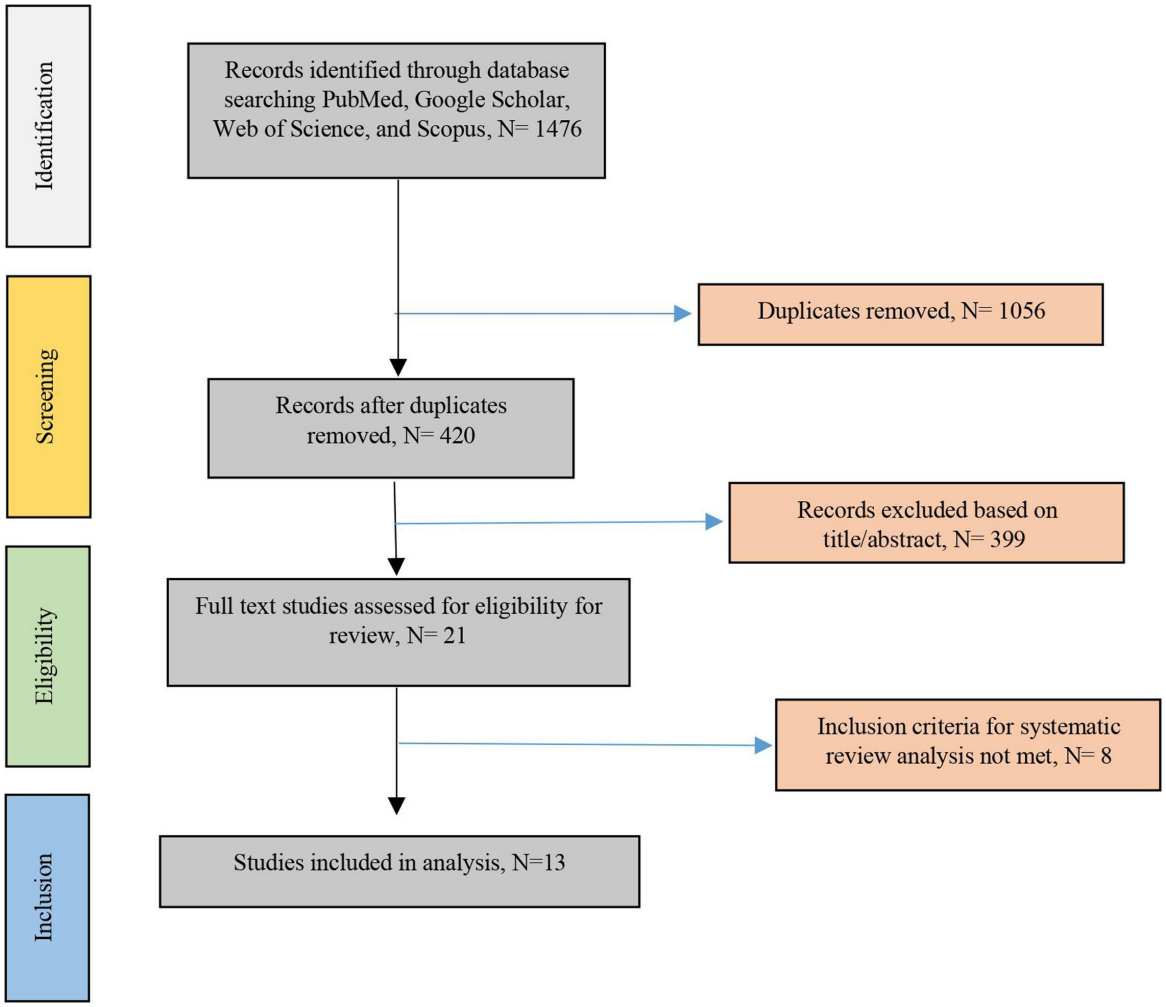
Flowchart of the study selection process.

The extensive literature review combined with a manual search resulted in a total of 1,476 studies. After removing 1,056 duplicate entries, 420 articles remained for investigation. A thorough screening of titles and abstracts led to the identification of 13 pertinent studies, discarding those that were not relevant. Five full-text articles were excluded from the analysis due to being published in non-English and three versions were also excluded from the analysis due to being not full-text available. Ultimately, the authors thoroughly examined the complete texts of the 13 selected studies released through September 2024. The process of study selection is illustrated in Figure 1, following the guidelines outlined by the Preferred Reporting Items for Systematic Reviews and Meta-Analyses Protocol in a flow diagram.

### Individual studies

The reviewed studies aimed to provide insights into the methods used in adapting and validating the CERQ. A concise overview of the methods used in each study is presented below, organized chronologically by publication year. This overview is based on various criteria, including the research year, authors, alterations made, involved professionals, publication specifics, location, language of adaptation, and the demographics of the study population, all of which are summarized in Table 1. Detailed explanations of the adaptation processes and the examination of psychometric properties will follow in the subsequent sections.

**Table 1:** Descriptions of the included studies.

	Language	Author(s)	Publication year	Journal	Participants
1	English	Garnefski et al.	2000	Personality and Individual Differences	*N* = 547 547 high school youngsters
2	French	Jermann et al.	2006	European Journal of Psychological Assessment	*N* = 224 224 young adults
3	Chinese	Zhu et al.	2008	Psychology Press	*N* = 791 791 participants from two universities
4	Spanish	Domínguez-Sánchez et al.	2011	Assessment	*N* = 615 615 students
5	Turkish	Tuna et al.	2012	Journal of Psychopathology and Behavioral Assessment	*N* = 396 396 participants from several universities
6	Persian	Abdi et al.	2012	Procedia - Social and Behavioral Sciences	*N* = 503 503 university students
7	Japanese	Sakakibara et al.	2015	Japanese Psychological Research	*N* = 324 324 volunteer under-graduates from three universities
8	Brazilian	Schäfer et al.	2018	Trends in Psychiatry and Psychotherapy	*N* = 445 445 university students
9	Arabic	Eissa Saad et al.	2020	International Journal of Psycho-Educational Sciences	*N* = 840 840 adolescents from six middle schools
10	Indonesian	Prastuti et al.	2020	Journal of Educational, Health and Community Psychology	*N* = 102 102 adults
11	Australian	Rice et al.	2022	Australian Psychologist	*N* = 781 781 Australian adults
12	Urdu	Shahzad et al.	2022	Frontiers in Psychiatry	*N* = 237 237 Male participants
13	Bangladeshi	Uzzaman et al.	2024	Journal of the Indian Academy of Applied Psychology	*N* = 1,000 1,000 participants

#### English

The original version of CERQ was developed by Garnefski et al. (2001). This questionnaire has items about how the cognitive process regulates emotions and how this may affect emotional development. Developers conducted a test–retest design and also performed the principal component analysis. After analyzing, the test–retest reliability of the subscale was adequate to good and they found that cognitive coping strategies are more relevant to negative experiences such as depression and anxiety (Garnefski et al., 2001; Garnefski and Kraaij, 2007).

#### French

The French adaptation of the CERQ was conducted using the back-translation method. Initially, a bilingual individual fluent in both French and English translated the original English version into French (Jermann et al., 2006). Subsequently, another bilingual person translated this French version back into English. Any differences that arose during this back-translation process were reviewed, and necessary modifications were made to the French version of the CERQ (Jermann et al., 2006).

#### Chinese

The Chinese version of CERQ was performed utilizing the back-translation technique (Zhu et al., 2008). Initially, a translator translated the original texts from English to Chinese. Then, another translator translated the Chinese version back into English. Finally, the original CERQ version was assessed against the back-translation. In cases where inconsistencies were identified in the back-translation, the translators collaborated to refine and correct the Chinese version (Zhu et al., 2008).

#### Spanish

The Spanish adaptation of the questionnaire, known as the CERQ-S, was crafted following the standards set by the International Test Commission and implemented using a back-translation approach based on the initial English version (Domínguez-Sánchez et al., 2013; Hernández et al., 2020). The process was performed in three key steps: (Grecucci et al., 2020) a team of bilingual psychologists translated the original English version into Spanish; (Fathipour-Azar and Khalafbeigi, 2021) a separate bilingual psychologist then translated the Spanish version, CERQ-S, back into English; and (Shahzad et al., 2022) any inconsistencies identified during this process were thoroughly reviewed and the necessary adjustments were applied to ensure the accuracy of the CERQ-S (Domínguez-Sánchez et al., 2013).

#### Turkish

The English version of CERQ was translated into Turkish by three independent clinical psychologists proficient in both English and Turkish (Tuna and Bozo, 2012). The original CERQ was then compared with the back-translated version by two psychology professors. After reviewing the translations, necessary modifications were made before finalizing the Turkish version. Ultimately, an independent translator conducted a back-translation into English (Tuna and Bozo, 2012).

#### Persian

The Persian version of the CERQ underwent a meticulous back-translation process (Abdi et al., 2012). Initially, two bilingual psychologists proficient in both Persian and English translated the English CERQ into Persian. Subsequently, a third psychologist, also fluent in both languages, conducted a back-translation of the Persian questionnaire into English. Any inconsistencies identified during this back-translation were thoroughly reviewed, leading to necessary revisions of the Persian CERQ to ensure its accuracy and clarity (Abdi et al., 2012).

#### Brazilian

The CERQ’s English version was translated into Portuguese by two independent translators proficient in both languages (Schäfer et al., 2018). Afterwards, two additional independent translators carried out a back-translation into English. Three psychologists then reviewed the original English version alongside the back-translated versions to identify the items that most accurately reflected the originals. These selected items formed the basis of the preliminary Brazilian version of the CERQ. Finally, four expert judges with specializations in emotion regulation, human cognition, and psychometrics assessed this preliminary version (Schäfer et al., 2018).

#### Indonesian

The forward translation process was performed in the following manner. Two translators converted the CERQ into Indonesian. Subsequently, a professional English translator re-translated the Indonesian version of the CERQ tool. The outcomes of this translation were evaluated through collaborative discussions in the expert panel. Also, assessments were carried out by three experts who possessed proficiency in English and a thorough understanding of the translation construct (Prastuti et al., 2020).

#### Urdu

The instrument was initially translated into Urdu by two independent translators (Shahzad et al., 2022). They were given comprehensive information about the scale content, the study goals, and details regarding the target sample to assist them in accurately translating the original English version of the CERQ into Urdu. Once the initial translations were completed, the instructions, items, and formats of the two Urdu versions were meticulously compared with one another, as well as with the original English scale, by an expert panel. The finalized forward-translated version was subsequently forwarded to two additional expert translators for back-translation. These individuals had no involvement in the initial translation process and were completely unaware of the original CERQ, thereby reducing potential bias in the back-translation process (Shahzad et al., 2022).

#### Bangladeshi

Initially, the CERQ was translated into the Bangladeshi version, followed by a thorough review and revision. Subsequently, a panel of six translation experts evaluated the translation and made corrections and corrections as needed (Uzzaman et al., 2024).

### Rating procedure

The administration process of various versions was quite simple. Participants completed the questionnaires either in person or via postal mail, carefully considering each item and selecting the response that best reflected cognitive strategies for emotion regulation. The CERQ is a 36-item scale based on the Likert format, featuring five response options that range from ‘almost never’ (Grecucci et al., 2020) to ‘almost always’ (Kraft et al., 2024). To determine higher and lower adaptive scores, the scores of all relevant items are added together. The high adaptive component consists of five subscales: acceptance, positive refocusing, planning refocuses, positive reappraisal, and putting things into perspective. In contrast, the less adaptive strategies encompass four subscales: self-blame, rumination, catastrophizing, and blaming others. The total CERQ score is calculated by aggregating the scores from all 36 items, with a possible score ranging from 36 to 180. Subscale scores are derived by summing the relevant items within each subscale, with each subscale yielding a score ranging from 4 to 20 (Shahzad et al., 2022; Uzzaman et al., 2024).

### Risk of bias

The 13 versions selected in the method section were subjected to a comprehensive critical assessment, the specifics of which are outlined for each study in the results section. Table 2 summarizes these evaluations.

**Table 2:** Descriptions of the translation process.

	Language	Forward translation	Expert panel	Back translation	Pilot study
1	French	1 translator	−	1 translator	−
2	Chinese	1 translator	−	1 translator	−
3	Spanish	a bilingual group expert in psychology	−	1 bilingual psychologist	−
4	Turkish	3 independent graduate clinical psychology students	2 psychology professors	1 translator	−
5	Persian	2 bilingual psychologists	−	1 bilingual psychologists	−
6	Japanese	−	−	−	−
7	Brazilian	2 translators	3 psychologists	2 translators	+
8	Arabic	−	−	−	−
9	Indonesian	2 translators	1 professor and 2 doctors of Psychology	1 translator	+
10	Australian	−	−	−	−
11	Urdu	2 bilingual clinical psychologists	4 bilingual clinical psychologists	2 bilingual clinical psychologists	−
12	Bangladeshi	+	6 translators	+	+

## Discussion

According to the literature review, self-assessed questionnaires are more adept at illustrating the effects of disorders than many other forms of evaluation. One of the most widely used in the field of cognitive and emotional control is CERQ (Jermann et al., 2006). Since its publication, the CERQ has gained considerable traction in clinical and research sectors due to its effectiveness (Uzzaman et al., 2024). The questionnaire takes less than 10 minutes to complete, making it less time-consuming than alternative assessments (Garnefski and Kraaij, 2007). In recent years, there has been a noticeable rise in publications that have adapted the English version of the CERQ into various regional languages. This practicality establishes it as an effective tool for providing targeted cognitive strategies for emotion regulation, particularly in managing stress (Tyra et al., 2024). This systematic review included 13 translated versions of the questionnaire. The adaptation process in all versions was not a direct translation of the English version and also underwent cultural and linguistic alignment. Alongside this, the validation details of various versions were investigated. The review of CERQ versions indicated that only three versions had pilot or pre-testing studies including Brazilian, Indonesian, and Bangladeshi.

Numerous studies have examined the relationship between CERQ and a range of assessments, uncovering noteworthy relationships across diverse populations and research approaches (Tyra et al., 2024). These studies have shown a strong correlation between the adapted CERQ with other questionnaires or symptoms of depression and anxiety including Symptom Checklist-90 (SCL-90), Beck’s Depression Inventory (BDI), Revised Beck Depression Inventory (BDI-II), State–Trait Anxiety Inventory–Trait version (STAI-T), State–Trait Anger Expression Inventory-2 (STAXI-2), Positive And Negative Affect Schedule (PANAS-PA), Positive And Negative Affect Schedule (PANAS-NA), Self-Efficacy Scale of Wong and Law Emotional Intelligence Scale (WLEIS), Depression Anxiety and Stress Scale 21 (DASS-21) and other mental health self-report scales (Knowles and Olatunji, 2020; Derogatis and Unger, 2010; Jackson-Koku, 2016; Petermann, 2016; Kühner et al., 2007; Bados et al., 2010; Crawford and Henry, 2004; Wong and Law, 2002; Lovibond and Lovibond, 1995). The recent findings indicated a relationship between socioeconomic status and CERQ scores, revealing differences among diverse socioeconomic groups (Antu and Bakul, 2023; Muñoz-Navarro et al., 2021). This challenging result suggests that individuals from different socioeconomic backdrops may employ various cognitive strategies when faced with emotional challenges (Antu and Bakul, 2023).

Even though the main focus of this review is cultural adaptation methodology, an attempt was made to provide validity and reliability information and statistical findings and document a comprehensive understanding of the adapted versions of the CERQ.

### The translation and adaptation process

The CERQ has translated modifications and validation in various languages to assess cognitive strategies related to emotion regulation under stress (Tyra et al., 2024). The effective adaptation and validation of the CERQ for different languages and populations highlight the importance of consistently and reliably measuring these cognitive strategies. Traditional translation methods or “forward-backward approach” included a forward translation, back translation, and review (Gorecki et al., 2014). The predominant translation method identified in the literature reviewed was Brislin’s traditional back-translation model (Jones et al., 2001); however, it was often referenced without using Brislin’s name. In some published versions, there is detailed and supplementary information about all the stages of the study. A review of the various versions showed that most of them were translated by professionals. For example, psychologists translated the Spanish, Turkish, Persian, and Urdu versions, while translators handled the French, Chinese, Brazilian, and Indonesian versions (Prastuti et al., 2020; Jermann et al., 2006; Zhu et al., 2008; Schäfer et al., 2018). However, there is insufficient information regarding the Japanese, Australian, and Bangladeshi translations (Uzzaman et al., 2024; Urano et al., 2022; Rice et al., 2022). Additionally, only the Turkish version had more than two translators. To ensure the cultural adaptation of questionnaires, it is important to utilize expert panels. An expert panel was employed for the backward translation in the Turkish, Brazilian, Indonesian, and Urdu versions (Shahzad et al., 2022; Prastuti et al., 2020; Tuna and Bozo, 2012; Schäfer et al., 2018).

### Validity

Although face and content validity has been assessed for most languages, the findings have not been thoroughly detailed, and do not present statistical results. The construct validity of the adapted version has been established in multiple studies conducted in various languages, except Brazilian and Bangladeshi (Uzzaman et al., 2024; Schäfer et al., 2018). Each version has shown a correlation with different scales and tools, yet only the Turkish and Japanese versions demonstrated a correlation with the self-efficacy scale (Tuna and Bozo, 2012; Sakakibara and Endo, 2016). The Pearson correlation coefficient also was reported only in the original English version (*r* = 0.42) (Garnefski et al., 2001). Table 3 presents the validity types and properties. The structural validity model was reported to be a good fit in all versions except Persian and Urdu.

**Table 3:** Validity and reliability of translated versions of CERQ across languages.

	Language	Construct validity	Total internal consistency (Cronbach’s alpha coefficient)	Test–retest reliability	Subscale internal consistency (Cronbach’s alpha coefficient)	Pearson correlation coefficient
1	English	SCL-90 measures in depression and anxiety section Anxiety: Partial Depression: Significant	0.92	Positive: 0.62 Negative: 0.62 Cognitive: 0.64	-	0.42
2	French	BDI-II: positively correlated	-	-	0.68–0.87	-
3	Chinese	Correlated with symptoms of depression and anxiety: depression: positively correlated anxiety: positively correlated	0.79	Total: 0.64 Subscales: 0.44–0.78	0.76–0.90	-
4	Spanish	BDI: positively correlated STAI-T: positively correlated STAXI-2-T: positively correlated PANAS-PA: positively correlated PANAS-NA: positively correlated	-	0.49–0.73	0.61–0.89	-
5	Turkish	Self-Efficacy Scale: positively correlated	-	0.50–0.70	0.72–0.83	-
6	Persian	acceptable construct validity	-	-	0.64–0.82	-
7	Japanese	Self-Efficacy Scale: positively correlated	0.72–0.83	0.50–0.70	0.44–0.57	-
8	Brazilian	-	0.70	-	0.71–0.88	-
9	Arabic	Wong and Law Emotional Intelligence Scale: positively correlated	-	0.92	-	-
10	Indonesian	high construct validity with high Composite Reliability (CR)	0.79	-	0.70–1.00	-
11	Australian	DASS-21: positively correlated Mental health self-report: positively correlated	-	-	0.79–0.91	-
12	Urdu	DASS-21: negatively correlated RSES: positively correlated	0.80	Total: 0.86 Subscales: 0.76–0.99	0.70–0.89	-
13	Bangladeshi	-	Adaptive CERQ: 0.85 Less ACERQ: 0.80	-	-	-

### Reliability

Inter-rater reliability was reported utilizing Intra Class Coefficients (ICC) and Pearson rank coefficients. Seven versions including English, Chinese, Japanese, Brazilian, Indonesian, Urdu, and Bangladeshi demonstrated internal consistency for the overall CERQ (Shahzad et al., 2022; Prastuti et al., 2020; Garnefski et al., 2001; Uzzaman et al., 2024; Zhu et al., 2008; Schäfer et al., 2018; Urano et al., 2022; Sakakibara and Endo, 2016). However, except for the English, Arabic, and Bangladeshi versions, the other translations showed good internal consistency for their subscales (Garnefski et al., 2001; Uzzaman et al., 2024; Saad and Kamel, 2020). In addition, seven English, Chinese, Spanish, Turkish, Japanese, Arabic, and Urdu versions reported evidence of test–retest reliability (Shahzad et al., 2022; Garnefski et al., 2001; Tuna and Bozo, 2012; Domínguez-Sánchez et al., 2013; Saad and Kamel, 2020; Zhu et al., 2008; Sakakibara and Endo, 2016) (See Table 3).

The French and Brazilian versions have reported confirmatory factor analysis (CFA). By establishing cut-off points and evaluating psychometric properties, researchers and clinicians can effectively measure the impact of cognitive strategies on emotion regulation. These versions of the CERQ use a cut-off score of 0.95 (Jermann et al., 2006; Schäfer et al., 2018). Also, the sensitivity index is reported in the Australian version of the CERQ (Rice et al., 2022). This detailed information is reported in Table 4. The psychometric properties of each version were examined following the COSMIN guidelines. Consequently, we applied the updated COSMIN criteria for the various versions of the CERQ, as outlined in Table 5.

**Table 4:** Cut-off points of translated versions of CERQ across languages.

	Language	Validity	Reliability	Cut-off point	ROC	Sensitivity (%)	Specificity (%)
1	English	+	+	−	−	−	−
2	French	+	+	0.95	−	−	−
3	Chinese	+	+	−	−	−	−
4	Spanish	+	+	−	−	−	−
5	Turkish	+	+	−	−	−	−
6	Persian	+	+	−	−	−	−
7	Japanese	+	+	−	−	−	−
8	Brazilian	−	+	0.95	−	−	−
9	Arabic	+	+	−	−	−	−
10	Indonesian	+	+	−	−	−	−
11	Australian	+	+	−	−	+	−
12	Urdu	+	+	−	−	−	−
13	Bangladeshi	+	+	−	−	−	−

**Table 5:** Updated criteria of COSMIN guideline for assessment of the CERQ versions.

	Language	Structural validity	Internal consistency	Reliability	Measurement error	Hypotheses testing for construct validity	Cross-cultural validity\measurement invariance	Criterion validity	Responsiveness
1	English	+	+	−	?	+	?	?	?
2	French	+	?	?	?	+	?	?	?
3	Chinese	+	+	−	?	+	?	?	?
4	Spanish	+	?	−	?	+	?	?	?
5	Turkish	+	?	−	?	+	?	?	?
6	Persian	?	?	?	?	+	?	?	?
7	Japanese	?	+	−	?	+	?	?	?
8	Brazilian	+	+	?	?	?	?	?	?
9	Arabic	+	?	+	?	+	?	?	?
10	Indonesian	+	+	?	?	+	?	?	?
11	Australian	+	?	?	?	+	?	?	?
12	Urdu	?	+	+	?	+	?	?	?
13	Bangladeshi	+	+	?	?	?	?	?	?

If reported item was ≥ 0.70; +, Item not reported; ?, If reported item was < 0.70.

Additionally, the CERQ is a valuable scale that enables the identification of individuals who may need additional assessment for communication and participation problems. Therefore, its translation and cultural adaptation to other languages is recommended. The authors suggest areas for further investigation, such as exploring additional cultural contexts or populations to enhance the generalizability of the CERQ. Also, we recommend using the CERQ in clinical settings to assess emotional regulation strategies in various psychological interventions.

## Conclusion

This study presents the processes of translation, cultural adaptation, and psychometric properties using CERQ versions. For the correct translation process, there is a need to consider linguistic, structural, and technical equivalents in the translation process, which increases accuracy in a cultural adaptation approach. Also, this review offers a comprehensive examination of cross-cultural adaptation and psychometric evaluation, guiding future researchers in choosing the most effective adaptation and validation methods. Furthermore, the CERQ emerges as a crucial instrument for screening, facilitating the identification of individuals who may require further assessment or intervention for emotion regulation.

## Data Availability

The original contributions presented in the study are included in the article/supplementary material, further inquiries can be directed to the corresponding author.

## Footnotes

^1^ https://www.covidence.org/
